# A Memoir on the Radical Cure of Varices of the Inferior Extremities

**Published:** 1848-02

**Authors:** Thomas Rima

**Affiliations:** Venice


*A Memoir on the Radical Cure of Varices of the Inferior Extremities.* By Prof. THOMAS RIMA, of Venice. Translated from the Italian by JAMES BRYAN, M. D., Lecturer on Surgery, Philadelphia; Honorary Member of the Philadelphia Medical Society; Corresponding Member of the New York Medico-Chirurgical Society; formerly Professor of Surgery and Medical Jurisprudence in Castleton, Vt.; late Surgeon and Physician to the Philadelphia Dispensary, &c. &c., with additions by the translator.

In translating the following able memoir, with its predecessor, on varices, from the Italian, we were influenced by the desire of presenting to the American medical public, a treatise on a disease of great importance; and one, in the treatment of which, the profession has long been divided in opinion. The great experience and high standing of the learned author, a number of whose cases we had the pleasure of examining, in the course of treatment, in the great Civil Hospital of Venice, in 1838; the peculiarity of his views, and the apparent candour of the writer, induced us to suppose that we were performing a labour which would not be unacceptable to our medical brethren. The many difficulties which still surround this very common disease, with the want of success, indicated by the great variety of treatment adopted by different practitioners, seem to us to be good reasons for the further investigation of the subject, and a closer examination of the opinions and treatment of those whose experience entitle them to some consideration from our profession. The theory of Prof. Rima, which he defends so ably, is one, to say the least of it, different from what is ordinarily maintained, except, perhaps, to a very limited extent. If this theory be sustained by practice, we certainly have a very simple mode of curing a very troublesome disease.

With the hope of adding something to the general stock, we have appended a few remarks on a mode of treatment, adopted by us some 14 years ago, and which, with us, has been found successful. A private practice of some fourteen years, with the public practice in the Philadelphia Dispensary for several years, has afforded us the means of testing its value. We confess never to have operated on the plan of Prof. Rima, yet, as is well known, the plan was recommended by Celsus himself. "Igitur vena omnis, quae noxia est, aut adusta tabescit, aut manu exciditur."—*Lib.* 7, *c.* 31.

### VARICES OF THE INFERIOR EXTREMITIES.

It is the boast of the present century, that in the progress of surgery, the experimental spirit of its learned cultivators has even attempted the application of the ligature to the great arterial trunks within the parietes of the thorax and abdomen. Among the Italians who merit honourable mention, are Vacca of Pisa, Medora of Padua, and Bartolazzi of Forli. But the most prominent and ardent, was the Englishman, Astley Cooper, who, in June, 1817, did not hesitate to make an incision (in the case of Charles Hudson) in the umbilical region; penetrate beyond the intestines, wound a second time the peritoneum, lacerating it with the finger, and place a ligature on the great trunk of the descending aorta, half an inch above its bifurcation. He did not effect a cure; the patient survived the operation forty hours, and thus proved that surgeons are authorized to perform it. Pathological Anatomy has become, in our day, a torch to guide us to those surprising undertakings, which the ancients had neither the courage to conceive or to execute.

Cooper knew that Desault and others, had observed in autopsies the spontaneous obliteration of this great artery of the thorax, without causing the cessation of life. He, with greater reason, might look for this happy result in the abdomen, and be encouraged to attempt a dubious operation, where there was no other hope. "Melius est anceps quam nullum expiriri remedium." But experiments on the veins have not progressed with equal pace. In operating upon these, for the radical cure of varices, in the lower extremities, the most common, most inconvenient, if not the most dangerous seat of the disease, Scarpa, Monteggia, Baynton, the Ucelli, and many other distinguished practitioners, have signally failed. It was natural that such unfortunate results should discourage young surgeons; it being very certain that an operation performed by these luminaries of our art must have been well done.

The veins, although furnished with a muscular coat, in the same way as the arteries, are yet more fragile, and subject to very great distention. Their recision is not free from danger, on account of the lesion of nerves, which produces debility and severe pains, as occurs in bleeding in the feet.

When the veins become varicose, they undergo, so to speak, a change in their nature, and acquire the consistence of arteries. They then become more subject to phlogosis, which is extended with great rapidity, to the larger vessels, and through these, to the centre of the circulation, inducing death in a very few days.

In spite of this pathological alteration in the venous canals, an operation is not always contra-indicated; surgeons should then not resort to it with entire confidence. Illustrative reasoning on the proximate cause of varices, will induce us to operate, and a practice of more than a quarter of a century on this branch of clinical disease, with a comparison of the results, entitle me to give an opinion on the relative value of the various operations proposed.

The Civil Hospital of Venice, with its numerous patients, has afforded us, during the last sixteen years, numerous opportunities of adding to the facts which were gathered in the Military Hospital of Mantua, and the Civil Hospital of Ravenna. Chief of the service, and clinical professor for several years, at first in the great Military Hospital of Milan, I had an opportunity of practically approving the clinique of Home. Composed, as was the garrison of that capital, at that time, of the flower of the Franco-Italian army, it was very natural that varices should be found among those robust warriors, and that active labour was demanded of the surgeon.

Thirteen years afterwards, in order to become an ordinary member of the Venetian Atheneum, I wrote a letter (the 29th of December, 1825,) and added an article on the proximate cause of varices in the inferior extremities, and on their radical cure. We considered ourselves, even then, in a condition to prove, both by reasoning and facts, that it consisted in an inverse movement of the blood in the great saphena, which gravitated in column from above to below, from the centre to the extremity. That hence the valves were paralysed, and the venous tunics were, in various ways, debilitated.

Ignorant, moreover, of this fact, we operated according to the precepts of others, and the effect, in a great measure, corresponded.

But we have since learned, from our own experience, the true proximate cause of varices, as it is illustrated by various morbid phenomena, of which we will present a full account in speaking of and discussing the different modes of operating.

Among our many operations at that time, some remained completely cured; the cure of the varices was accompanied, in some cases, by the cicatrization of chronic ulcers on the legs; in others, there occurred only an improvement in them, and they were no longer troublesome.

It must not be concealed, that in Francesco Rosa, a pharmaceutist of Lonigo, aged 67 years, there was reason to fear the fearful consequences of phlebitis. Operating on the 6th of May, 1825, by recision, to the extent of half an inch, on the great saphena, on the inferior part of the right thigh, *the blood flowed freely from both extremities of the vein which was tied.* On the third day a slight erysipelatous inflammation showed itself along both trunks of the divided saphena, on which were formed successively three abscesses, two on the thigh, and one on the leg, which were opened by the knife. The vein was completely obliterated at the seat of the suppuration. The varicose enlargement had so entirely disappeared by the third day after the operation, that a complete and permanent cure was expected. In fact, nearly all the difficulty in locomotion, which had made the patient inactive for ten years, disappeared. A small elevation, apparently herpetic, which had at various times been located on the malleolus externus, became also cicatrized. The varicose tuberosities along the leg, which had made depressions on the anterior internal fascia of the tibia, became almost invisible in the horizontal position; they became turgid in the vertical, but presented only one-third their primary volume. Although the progress of the cure in the above case was retarded by the phlebitis, we cannot consider the result unfortunate, for we do not know that this accident was the necessary consequence of the operation. It is certain from this, that accidents may occur which cannot always be either foreseen or avoided. If sometimes these things happen, and impede the happy result of a rational operation, we are not justified by this in discarding the knife and giving the cases up to ulterior and more doubtful treatment.

At the time we wrote the above article, we had operated on twenty cases, and all with a result more or less favourable—the event varying only in degree. But it is very natural that we should not be successful in all our cases. During 14 years we have lost two patients by phlebitis.

On the 19th of June, 1827, Nicolo Tornat, a stage driver of Carniola, aged 43 years, died—it being the sixth day of the operation. The very largest varices occupied for 10 years his left thigh, and descended in a reticulated form over the whole leg; when obliged to move he could undergo no fatigue, and very little exercise when upon his feet. I cut through a transverse elevation of the integuments, in the middle of the right thigh, raised the vein with a pair of forceps, and divided to the extent of more than half an inch the indurated and thickened vein, with a pair of scissors. The blood flowed guttatim from the cut and tied extremities of the vessels. The post obit, performed by my assistant, Sig. Giovanni Battista Fabris, in Valdobiadine, exhibited the whole venous system inflamed. In both the cut and tied extremities, a small quantity of pus was found. Traces of arteritis existed in other parts of the sanguiferous system. The lungs and the heart were engorged with blood.

On the tenth day after ligation of the great saphena of Giovanni Rosetti of Burona, aged 58, a bargeman, in August, 1829, phlebitis was established. This, perhaps, was caused by his occupation, which, in future, will influence us as a contra-indication to the operation. There was developed in this case the most intense inflammation at the termination of the second day. The repeated emissions of blood, with both local and general sedatives, were insufficient to check the disease. Finally, the body came under the inspection of our chief assistant, Doctor Trevesini, acting J. R. fiscal physician. The whole venous system appeared indurated, not excepting the parietes of the heart. The saphena, the iliac and the cava, had decidedly increased in calibre, and consistence; the opening of the saphena had been obliterated at the point ligated, and was covered on the internal surface with plastic lymph. Nor was the brain unaffected, a slight extravasation being found in the lower ventricle.

It is natural that the necessity of recording in his catalogue the deaths of some of his patients should increase with every operator. But it is especially proper to do so, that he may publish the unfortunate as well as the fortunate cases, for the instruction of others. By the multiplication of facts alone, are practitioners enabled to form a good basis for a rational treatment. Two deaths out of twenty-five cases, more or less completely successful, are not sufficient to deter us from the practice, nor do we stop here in fact, the number having increased to 37.

Of which there were:

| | | |
|---|---|---|
| Radically cured of varices, | - - - | 8 |
| Do. with a scar, | - - - | 6 |
| Improved, | - - - - - - | 13 |
| With little amelioration, | - - - - | 6 |
| With no improvement, | - - - - - | 2 |
| Death, | - - - - - - - | 2 |
| Total, | | 37 |

This number isonly that of the patients—the operations were 41. In some cases the operation was repeated on the opposite vessel, in others it was repeated on the same vessel, when the first had not succeeded, and when the distribution of the veins was such as to induce us to expect a cure from an operation performed in another appropriate point.

The last operation, on the 15th of October, 1836, and which terminated on the 15th of December following, was performed on Natale Zecchinni, a countryman, residing in Campalto, aged 34 years. He had varices for three years on the thigh and left leg, besides a small ulcer over the internal malleolus, cured several times by rest, but reappearing immediately with motion. In this case, I excised a portion of the saphena, to the extent of half an inch above the knee. It proceeded as usual, but without much hæmorrhage; the blood came from each extremity of the vessel. The wounds healed by the first intention, with the application of adhesive plaster. The flow of blood was arrested by two compresses applied above and below the incision, sustained by a bandage. A few hours after, a sense of drawing in the wound indicated the existence of tumefaction, supervening on the operation. It ceased entirely on the relaxation of the adhesive strap. There remained on the third day only a slight fever, with a painful swelling under the wound. This disappeared immediately by the mere application of cold bathing. On the seventh day he complained much of hunger.

He was allowed a better diet; not contented with this, he committed certain dietetic misdemeanors. A very severe phlebitis was the consequence. On the tenth day a very active fever set in, preceded by four hours very cold chill, with but little remission. On the next day it returned, with but two hours chill. Great prostration of strength, moral and physical depression, vomiting, burning heat, insatiable thirst, oppressed respiration, bitter taste in the mouth, the tongue dry and furred, were the principal symptoms, united with constipation of the bowels. On the third day inflammation of the vein was very obvious, which appeared red under the integuments, painful to the touch, representing a serpentine line. On the fourth day the phlogosis had attacked the common integuments, from the seat of the incision up to the groin. At first the bitter salt, tartar emetic in small doses, gum arabic with calomel, continued for several days, repeated cathartic clysters, with refreshing or cooling drinks, were the medical treatment used. But the anchor of salvation was 160 leeches applied during a few days, keeping up, in the mean time, a continual stilicidium of blood. He got well. The veins of the leg were scarcely visible on the fourth day after the operation. Now they swell up about one-fourth of their former size, in the vertical position and on taking exercise. This case is cured with the exception of the swelling of the leg during locomotion. Nothing can be expected, according to our principles, for the veins of the thigh, yet even these were diminished two-thirds. We believe that this improvement may be referred to the phlebitis. Lying in bed with the knees a little higher than the hip, the blood did not reflow from above in the trunk of the saphena, the tunics being inflamed, contracted, in this way diminishing the diameter of the vein. It is to be hoped that this amelioration will continue to progress. The ulcer over the malleolus is cured. Supported for a time with buckskin, the leg will lose the disposition to swell in locomotion, and every thing indicates a permanent cure. The operation has restored an active labourer to his fields. Before bringing in the arguments which support our theory of the inverse movement of the blood, as the proximate cause of varices, it will not be out of place to speak here somewhat of the modes of operating, that useful deductions may be drawn for practice.

Scarpa and Vacca differed in opinion on the preference to be given to the temporary over the permanent ligature in the cure of aneurism. The idea of substituting an animal ligature, for thread or silk, originated with us, as the former, composed of material easily decomposed, may be left in the wound with impunity, under the control of the lymphatics. I put it to the test in a case of popliteal aneurism, in 1820; the cicatrix did not form until after the third week, but without any remains of the ligature in the wound. In the same way I applied the ligature in a case of varices, to the great saphena, in Vettore Bessoni, of Ravenna, on the 8th of June, 1821. The cicatrix was formed in twelve days. A lymphatic fluid continued to exude for eight days. We now think that no ligature is necessary. This operation was put, to the test, before Cooper's operation for aneurism, but I shall not speak further of it in treating of varices. I excised a portion of a vein in the vicinity of the knee, from Melandri Marco Antonio, aged 48 years. It was the first experiment made to determine whether pressure alone was sufficient to arrest the hæmorrhage. The result was fortunate. Melandri was happy in enjoying a complete cure of varices, and of an ulcer on the left leg.

Paletta had already operated by excision in 1815 and in 1817; Ghidella had also done it. From experience we concur entirely with Cartoni in saying that it is the most appropriate means of curing varices and their ulcers of the inferior extremities.

Brodie proposed passing a convex bistoury horizontally through an elevated fold of the integuments, and turning the knife with its edge to the vein to cut the latter in retracting the knife. By placing a small roll of cloth to keep separate, and compress tightly the divided extremities of the vein, he attempted to keep them from approximating, pressed together the fresh margins of each vessel, and induced in this way rapid adhesion, and avoided the introduction of atmospheric air, always dangerous when brought in contact with the internal coat of the veins. But this incision under the integuments, although it appears very plausible, is not free from objections. If the vein be adherent to the integuments, as is frequently the case in the leg, one runs the risk either of injuring the latter, or of incompletely dividing the former. In pressing upon the instrument, it is difficult to limit to a precise line the depth necessary to render the incisions complete, without dividing the aponeurosis, the muscular fibre, or even the periosteum, especially if the incision is made on the anterior internal surface of the tibia, where the vein is covered only by the integuments. Nor is the danger imaginary, as I had occasion to know, in the case of a lady of Zante, several years ago. I thought I had divided the varicose vein which coursed along the bend of the leg. But the introduction of the roller was not sufficient to keep the divided extremities separated; the original communication was very soon restored. Nevertheless, as the diameter of the vessel was not entirely restored, the blood did not descend so copiously into the lower part of the vein. The lady experienced some benefit from the operation, and I observed her walking in the streets of Venice with greater expedition than formerly. Yet I prefer that the cut should be made by dividing the skin, and excising a portion of the vein.

Ever since the days of Celsus recision has been in use for the cure of varices. Marius bore with Roman firmness the extirpation of several varices of one leg, but had not the courage to submit to a like operation on the other. It is surprising that in our day also, the practice of opening for two or three inches the varicose vein, and filling it with lint in order to change the canal into an impervious substance, has been resorted to! In not entirely dividing the vein, great risk is run, lest from the contact of the lint and the atmospheric air the phlebitis be extended to the larger vessels. This treatment, moreover, is not proper where the vessel is adherent to the integuments and to the periosteum; since, for reasons which have been given, it is better to select a place somewhat higher up and divide the vein completely. The whole of the curative measures of the ancients by fire or caustic are equally objectionable; the temporary application of pressure by means of pincers, by the pin suture, or by other measures which protract local stimulation, are objectionable, render the case uncertain and protracted, expose the patient to constant pains, and double the dangers of phlebitis.

The slight and almost no advantage heretofore obtained in the cure of varices, may be referred to the fact, that sufficient importance has not been attached to their efficient and proximate cause. Attention has been directed only to the effect. It is to this also, that we may refer the fact, that the advantages obtained from pressure applied to them by means of plates of lead, or a laced stocking, have been merely palliative. They were useful in as far as they afforded a support, a mechanical one, to the weak part of the vessel, but without the power to restore in the least its primitive elasticity and resistance, and without opposing effectually the column of blood, always prone to gravitate from above downwards, to move from within to without—from the centre to the periphery.

The effect of compression, in whatever way applied, may be compared to a truss reacting against the inguinal ring, impeding the descent of a hernia, but not with the same result.

The elasticity of the truss presents in the various movements of the body a constant resistance to the protrusion of viscera of any volume. Compression on the vessel, on the other hand, is uncertain; it cannot be made efficient in retarding a fluid like the blood, which may pass through the smallest sinuosities of the venous canal. In compression, the least change or relaxation of the ligature or lace stocking, allows the column of blood, always disposed to it, to descend by the power of gravity.

To prove that the venous blood, in producing varices, breaks partially the law of the circulation, we have adduced facts which appeared to us to be incontrovertible. These are observed before, during, and after the operation.

Unacquainted ourselves with this derangement of the circulation, we wrote simply the facts which occurred under our eyes, without appreciating their value, as have other surgical writers. Let us return a quarter of a century, to our first operations, when we could not have been deceived by theory, as we had not at that time formed one to defend. We have before stated, that in all the individuals afflicted with varices of any size, on the legs, it was easily seen in the change of posture of the body, from the horizontal to the vertical, that the blood flowed from the crural vein through the great saphena, into the ramifications of these vessels. The effect of the experiment is the more striking the weaker the vessels. It will also be more perceptible, if, before causing the patient to rise, the blood in the great saphena is made to flow from below upwards. Hoping to explain it, we have observed the phenomenon for several years. We obtained a confirmation of this from our colleagues on the 28th of August, 1825, on an occasion when our attention was directed by Sig. Scill. Adriana, bankers, to a chambermaid in the residence of Sig. Mattiu. It was confirmed unanimously; and may be verified by any one, since neither wounds nor any painful experiment is necessary. In this patient, as in the greater number of ladies who tie the leg below the knee, elevated, varicose veins, were observed above the point tied. In other cases they first appear below.

This is sufficient to establish a fact long since observed by surgeons, since nearly all perhaps have been called to stop the hæmorrhage from a varicose vein of the leg, when it bursted spontaneously. They contented themselves with applying compression over the lacerated point. They observed the character of the hæmorrhage, and may have seen that the blood flowed from the superior trunk.

We must remember, finally, in order to render the operation effectual, to select that position of the limb which is convenient both to patient and operator, and in which the veins to be operated upon by incision or ligature, will be most turgid and prominent. If there be no perversion of the circulation, the horizontal position will be the most convenient. By compression with a finger on the superior portion, we may arrest the column of blood between the point compressed and the first valve below. But it is necessary, according to Volpi, to apply a ligature around the whole thigh, because all the superficial and deep seated vascular system arrests the natural ascent of the blood; hence, although we by this means induce an enlargement of the saphena vein, yet the tumefaction is limited and less than that obtained by the simple vertical position of the limb.

Home observed this fact, but without giving it its full importance. He thus writes: "It is a fact, that the veins are most enlarged when the patient stands upon his feet;" he made the patient sit on a stool placed upon a table.

He thus clearly perceived, that by inverting the natural arrangement of the circulation, the blood by the laws of gravity descended and enlarged the veins; in this way making them much easier to be operated upon.

Let us follow the surgeons, and we will see that even during the operation circumstances are developed which coincide with the above named facts, in giving new force and new support to our theory.

Let us refer, in the first place, to 1811, in Volpi's work, p. 162. He says, we operated on an old gentleman, Tanghetti, by incision, and afterwards by ligature on the great saphena, on the inside of the knee; the superior trunk, not tied, continued to throw out blood in a stream. It is known, even to those unacquainted with anatomy, that the venous circulation courses from the periphery to the centre, from the extremities to the heart. Now, to proceed from the thigh to the leg, there was proof in the case of Tanghetti that the blood moved in an opposite direction to that established by nature. Nor is there any reason to believe that there was any secondary branch which supplied the saphena above the incision; for he observes, it was isolated and very superficial, and by pressure applied with the finger, under Poupart's arch, the blood was entirely arrested. It was hence certain that the blood flowed from the crural vein, and he might, perhaps, have added, from the iliac and vena cava.

A little further on, Volpi writes, the observation of Sig. Rima has been confirmed in this, as well as in the other two cases, viz.: that the portion of vein below the ligature did not tumefy, as at first view might have been expected. If it be true, as has been stated, that the tumefaction of the varicose veins on the leg is produced by the reflux of blood from the larger trunk, the communication being cut off by the ligature, the fluid cannot have access to enlarge them, and the blood which ascends from the feet, passed through the smaller saphena to the popliteal and crural, proceeding in the direction produced by pressure on the descending column of the great saphena.

The operation performed upon Alesandrini, reported on page 140, militates most against our theory. He was affected with large varices on the dorsum of the foot and on the inside of the left leg. On the upper part they were as large as a finger, of which a group were seated near the groin; he suffered so much weight and pain in the leg, that he could not stand long upon his feet. Towards the end of September, 1811, the operation was performed on the inferior third of the leg, where the principle branch of the saphena presented an isolated portion. Not suspecting at that time the inverse movement of the blood, I selected an inferior, isolated part of the vein, to apply the ligature. If, then, we follow out the old theory, we would conclude that the nearer the foot we operate the better, as the vessels above will be the better protected. Being now better informed, we may understand (as Volpi has shown) why "the group of veins near the groin remained in their former condition," and why the varicose veins of the inferior part of the leg and dorsum of the foot were not inconsiderably diminished.

"The Alexandrian obtained an improvement inl ocomotion, but not so much as the others operated upon. The blood continued to flow back from above, and enlarged the veins, which were above the ligature. It was decided to operate again at a higher point. The trunk of the saphena presenting itself, isolated from the neighbouring branches, at a point near the knee, for the purpose of obtaining a more complete cure, Sig. Rima decided to apply at this place a new ligature. The result in a great measure, but not entirely, corresponded to his expectation. The varices on the leg were considerably diminished. But in the vicinity of the knee, rather more above than below the ligature, there remained an elevated, discreet, varicose group of veins; these, however, continued to subside, and became less tumid than those near the groin."

The history of the Alexandrian affords a conclusive proof in favour of our theory.

1. The ramifications of the veins below the ligature were not, and could not, be any more enlarged. The blood which descended through them from the great saphena, found in the ligature an insuperable obstacle.

2. This obstruction being placed on the natural passage of the circulation, the return of the blood must take place through the capillaries. It was then forced to pass by the anastomosis of the internal vessels of the leg, and be conducted successively from the femoral to the iliac and thence to the heart. Thus, previous to the application of the ligature, the course of the column of blood had already been reversed, and thus the new course had not only been made, but enlarged. Hence, little or no force was necessary to induce it to alter its primitive direction. Before the veins were varicose, pressure applied to the vein would induce an enlargement of the smaller inferior branches.

3. The blood which remained almost stagnant, between the last anastomosis and the ligature, could only by degrees be restored to the circulation. It had not gravitated sufficiently to force back the lower column, which assumed but slowly its new direction, for the purpose of being conducted, *de novo*, from the extremities to the centre.

4. The lymphatic vessels also assist in diminishing the stagnant mass below the ligature. That which has become condensed by coagulation is transformed into an inorganic mass, and becomes an integral portion of the living being. At the same time the various small varicose vessels, by virtue of their organic elasticity, continue to contract to their normal calibre, as is seen to take place in the uterus after gestation.

5. We are not to expect so much from the varices situated on the limbs above the ligature, because there is not in the vicinity of the groin an obliteration of the vein, which shall impede the blood disposed to yield to physiological laws, and by gravity descend from the crural vein. This is proved in the statement, "that the varicose group of veins always continued in the vicinity of the groin." They remain stationary, if they do not increase, as is said by Volpi to have occurred to himself in the following case, where he speaks of Pietro Capello, operated upon in the hospital of Pavia. He incised and tied the varicose veins on the malleolus. This very painful operation was followed by gangrene in the parts. Volpi, like ourselves at one time, and all surgeons, fell into this error. Not suspecting the inverse movement of the blood, it was supposed (and the supposition was rational) that the lower and nearer the foot, the more useful the obliteration of the vein in the cure of varices. Facts and reasoning did not accord with the supposition, but have demonstrated the opposite, as without knowing it the above operation has done. "We undervalue," says he, "(after the operation,) the rete varicosa which is found on the internal margin of the foot, extending from the internal malleolus to the sole of the foot, and to cure which it is necessary to operate upon the vein near the calf of the leg. All that space on the internal side of the leg, where the varices run in a varied serpentine direction, coursing along its whole extent, and extending to the thigh, and even to the groin, appears from the first much tumefied, and knots are formed on the external surface of the leg, which do not exist at first."

He could not in a clearer manner demonstrate that the cure of the varices, situated below the ligature or the obliteration, is followed by the cessation of a sense of weight in the course of the descending column of blood, but that those situated above the ligature become worse, and continue to experience a painful sensation of weight from the same cause. That which surprises me most is the false deduction which this writer draws from these facts: "taking for granted that in the cases in which—on account of the dilatation of the saphena—the blood flows back from the crural, as appears from the case reported by Sig. Rima himself, I should propose, in treating a leg in which the internal saphena was varicose, applying a ligature at the internal malleolus, and two or three along the leg." We think quite the contrary. We think it sufficient, so to speak, to place but one ligature above the varicosed parts; or, in other words, to obliterate by excision the saphena at a point above the varices, but not higher than they extend, since a lesion of the vascular system is always more dangerous in proportion to its vicinity to the heart, the common centre of the circulation.

Determined to take advantage of every circumstance calculated to elucidate the question, "whether the inverse movement of the blood be the true proximate cause of varices," we have collected numerous cases since the time of our article of December 29th, 1825, but it would swell our memoir too much, and render it uselessly prolix to adduce them. If the results of the treatment have not been completely successful, nothing has occurred which has not confirmed and substantiated the original idea. To obtain the whole, we should record what has taken place after the operation. There is one fact which we consider important and decisive, and it may be verified by any one who desires it.

The subject of this was Marmei Benedetto, a Venetian, aged 32 years, at present a seller of grains—at first in the vicinity of St. Giovanni e Paolo, afterwards near St. Antonino. For several years, in his youth, he was afflicted very much in locomotion by large varices, which occupied the left leg and thigh, which was hence much disposed to swell. He begged medical assistance in our hospital; was operated upon on the 13th of May, 1829, by the recision of half an inch of the varicose saphena, after the incision of the integuments. The blood flowed with its usual rapidity from the divided extremities of the vessel, but was easily arrested by the application of a simple circular roller and a compress along the divided vein. The varicose enlargement of the lower part of the leg disappeared completely. But there remained, and seemed to enlarge, a varicose fascia, which occupied the inferior third of the thigh. It was clear, according to the principles stated, that this phenomenon could not exist otherwise than by means of the blood which flowed from the femoral, by an inverse movement, and distended the saphena above the incision. To cure this, it was necessary to perform a second operation a little above the varicose fascia. It was done on the 25th of the same month. A complete cure crowned the resignation of the patient and the perseverance of the operator, who has frequently had occasion to see Marmei, and to receive with real pleasure his acknowledgment of gratitude, a compensation sufficient to efface the ingratitude of so many others.

All the established truths known should tend to correct the doubts and objections of the time. The present has its own. The anatomists and the physiologists send forth their anathemas. But supported by the observation of facts, we will be sustained by pathologists and clinical teachers. Should it be proved that we are in error, we will be grateful to him who will show the deception.

In looking over the various surgical works, it appears that several writers have thrown light upon this question. Many have described it as an extraordinary fact, and some have asserted that the true cause of varices consists in this partial perversion of the circulation.

Piorry writes in the "Journal Hebdomadaire," I know not to what extent the reflex movement of the blood in the veins may proceed. We often see in the varices of the inferior extremities something similar. Even when the limb is placed in a horizontal position, the valves do not impede the reflux of the blood. It would be interesting to see in persons affected with varices of the leg, and in whom this retrograde movement exists, whether there are any obstacles to the return of the blood to the heart.

Monteggia, who is most accurate in registering his own and recording the practical observations of others, joins us in pronouncing that as the proximate cause of varices, which we think we have proved and shown to a demonstration. "Why," says he, "in stopping for ever, by ligature, the course of the blood, do we not increase the tumefaction of the inferior veins, which receive the blood from the arteries, and have this trunk alone to empty themselves into. It must be that a great cause of the production and continuance of varices exists in the weight of the superior column of blood which gravitates to the inferior veins, in spite of the support of the valves, and in removing which (by the ligature) we remove the principal cause of their distension, by relieving them from the effect of gravitation. In fact, (continues he,) I have proved this; for the ligature is hardly applied, before the varicose veins which are below diminish and become soft, instead of becoming distended and full." Although doubtful, yet he describes what really takes place.

The remote cause of varices deserves now a brief examination. We must bear in mind that it is admitted that extremes meet; a paradox which teaches, that in proportion to the inertia, so will the surplus activity tend to produce the same effect—varices. Sometimes there exists in the sanguiferous system a natural predisposition to this. In one who lives an easy and sedentary life, the force of the venous impulsion is broken, even to the primary capillary attraction; the circulation becomes somewhat dependent upon the muscles to overcome the resistance of its own weight. This is increased at the confluence of the smaller vessels with the principal trunk. The fluid passing from a narrow tube into one larger, loses its velocity. If we consider an old man, we find that the energy of the venous system diminishes in spite of the increase of the antagonistic force. The effect of such a condition is the debility of the venous coats opposite to the valves; or the valves themselves offer but little resistance to the weight of the column of blood, which proceeds towards the heart very slowly. Commencing to yield, the venous coats present the first rudiments of varices. The application of heat in some cases developes them; it diminishes the resistance of the tissue, while the expansion of the circulating fluid tends to make it occupy more space, and thus increase the diameter of the canal. In this way, to the first varix a second succeeds, and a third progressing variously, and leaving even some parts in their normal condition. The varied gradations of debility give a nodose, unequal, and a somewhat serpentine form and direction to the diseased vessel. The valves are paralyzed, and like a weakened band, do not force the blood to continue in its natural course, but permit it to yield to the laws of hydraulics, and run wherever a declivity invites.

In order to give a proper explanation of the opposing cause, that is to say, where the excess of activity produces this effect, the varix, we must be governed by the pathological idea already expressed, on the true function of the valves. It is known that those veins alone which accompany the muscles destined to voluntary motion, are provided with them. Under the strong and frequent contractions of the muscles, the blood is compressed within the vein, and starts in two opposite directions. One column goes towards the heart, having in this mechanism acquired new velocity; the other is driven against the parieties and towards the first valve. It receives from the elasticity of both an impulse, which drives it with greater force in the direction of the circulation. As long as the impulse of this action and reaction (which may be termed the temporary sistole and diastole of the veins) is maintained in equilibrio, the celerity of the circulation is increased, and is maintained in a normal condition. But when, as occurs in the labouring classes, the blood is driven too long, and with too much force towards the valve, its elasticity does not react to the often repeated vibration. It yields to the pressure, from above downwards, of the sanguine mass. Thus we have the resistence weakened in the parietes of the vessels, and in the manner already indicated we have varices formed.

We have thus repeated the arguments which go to prove that varices may be produced by a force acting from the periphery towards the centre. From these facts, we learn that the blood may be driven with force from the smallest vessels into the large ones, whatever obstacle opposes, that it has power to overcome the tenacity of the vascular coats, and dilate them uniformly, as in an injection, not in knots; whilst the valves are, to a certain extent, folded over the internal superfices of the veins, and thus present an obstruction to the natural and progressive course of the blood.

I know not whether the form itself of the varices will add any value to the conjecture. We have not as yet made it a matter of observation. Operating on the 15th of October, 1836, on Natale Zecchini, a robust stage driver of Campalto, on various forms of varices, we observed that the arches were generally curved with the convexity to the side, or downwards, while the concave surface was in opposite directions. If such a direction of the curves were constant, or even frequent, it would be a material proof that the debility is produced by a force which gravitates from above downwards. Without reference to an operation, it would be worth while for some one to verify the observation. A fact to the contrary would not be sufficient to exclude the probability; since should the group of varices have existed some time, it may have undergone some change, either in form or direction.

Finally, a single reflection will suffice to conclude our memoir. Varix has always been considered a passive debility of the veins, an absolute atony of the membranous tissue. We are convinced that this pathological condition may be considered in two aspects. It is convenient to admit that there exists a simple dilation of the vein, and a proportionate diminution of the vascular tissue. This does not occur except on the tibia where the vein courses over the white aponeurotic tissues, separated from the muscles. When they are in contact with the muscular tissue and become varicose, long before the thinning of the tunics is observed, the increase in their size and thickness, which they preserve in proportion to their calibre, is manifested.

We have already stated that a portion of vein thus enlarged, cannot be distinguished from a portion of an artery. This was rendered obvious to our young surgeons, when we excised about an inch from the patient Natale Zecchini.

In proportion to the increased thinness, will be the increased diameter of the vein by the separation of the elementary molecules of which it is composed. This appears to resemble the fissures and cracks which are seen on the mammæ of some puerperal women, and better on the abdomen of parturient females. In some way there occurs a solution of continuity in the living organism; this proves a stimulus, and induces an afflux of fluids, a semi-phlogosis. There is consequently thrown out, plastic lymph which is spread through the intermediate cellular tissue of the two membranes, and thus increases their thickness.

We should consider what really takes place in the varicose veins which run over the white tissues of the leg, separated from the muscular tissue. Here the thinning of the membranes of the varix is such as to induce a hæmorrhage, which is inconvenient, if not dangerous. These different conditions of the veins, enlargement and thickening, are, in our opinion, the consequence of the presence or absence of muscles. Every one knows, that all the reproduction of animals depends upon the arteries. Muscular tissue abounds in these, and is also found in the venous coverings. Here, from the excess of stimulus, is thrown out the plasma, which increases the substance of the coats in proportion to the extent to which the varicose vessel has enlarged.

Matters proceed differently on the skin of the leg. The white tissues are not so abundantly supplied with arteries as to supply the elements of assimilation. The blood which would be carried to the vascular tunics, for their nutrition, passes directly to the smaller arterial ramifications. In proportion as this sanguineous irrigation is thwarted, the antagonistic power of the lymphatics to absorb the vascular tunics, and the integuments pressed upon from within to without, is increased. Nitches are, not unfrequently, formed in the bones in which the varix is placed. The integuments here also yield, like the vein; they become thinner and thinner, and ulcerate ; the weight of the column of blood finally overcomes the resistance of the soft parts, effecting the laceration of the vein and the integuments, and producing hæmorrhage.

Pathological anatomy will throw new light on the proximate cause of varices, illustrating better this branch of practical surgery, and rectifying the indication for their radical cure.

—

Taking for granted, that whatever the original cause of the disease (varices) may have been, the coats of the vessels were in an inflamed condition, and that the change in texture and density, which had taken place, was kept up by the presence of the blood in contact with them, and that, as is the case in other inflamed tissues, the blood itself was a stimulant to morbid action, and that the pressure outwards of the larger sanguine columns necessarily distended the vessels and kept up the irritation ; we supposed that in the treatment of enlarged veins, the following indications were to be met :

1st. The blood should, as far as possible, be kept out of the veins.

2d. The inflammation in their coats should be removed ; and

3d. An action in the tissues should be induced, which would result in the absorption of the abnormal deposits.

To meet the first indication, pressure by means of a well applied roller or adhesive straps, or both, should be resorted to. This pressure to be applied to the whole of the parts affected. The ulcers which often exist, and depend upon the disease of the veins, to be subjected also to pressure and treated with mild unirritating unguents.

The second indication to be met, by depletion, general, or local, or both, together with the use of cathartics, saline, or mercurial, as the case may demand. The use of cold water, and salt and water to the inflamed parts, with rest and the horizontal position.

The third indication, viz.: the removal of the abnormal deposits, which have taken place in the tissues of the vessels, to be effected by the use of mercurial ointment, frequently and well rubbed in.

These means were resorted to in a large number of cases, and were generally successful, I may say always, where the treatment was faithfully carried out by patient and surgeon.

The following case will illustrate the treatment according to this plan.

J. B., a strong, well made and laborious blacksmith, aged 45 years, applied to the surgical clinique of the Dispensary for relief, from varices of both legs, accompanied with two ulcers on the left one. The veins were very much enlarged and knotted, as large as my fingers, and not only involved both saphenæ on the legs, but extended up over the greater part of the thighs. Numerous cicatrices on each side of the tibiæ indicated ulcerative disease, and the former existence of ulcers. The disease, according to the patient, had existed seven years. He applied for relief on account of the pain on locomotion and general tumefaction of the limbs. Considerable febrile action existed, the tongue was furred and digestion impaired.

He was ordered to be bled freely, and take two compound cathartic pills; salt water to be applied to the extremities, and a roller from the toes to above the knees neatly applied. The ulcers were dressed with simple cerate and straps of adhesive plaster carefully placed over them and around the limb.

On the third day after, the patient presented himself at the clinique much improved; action on the bowels had been kept up by one pill each evening, and the salt water had been applied morning and evening, followed by the adhesive straps and a muslin bandage. He was directed two ounces of mercurial ointment, a little simple cerate, and an ounce of sulphate of magnesia. The ointment to be applied morning and evening freely over the whole of the diseased parts, after the use of the cold salt and water, and drying the limb with a coarse towel. The ulcer to be dressed as before. A table spoonful of the sulphate of magnesia to be taken every other evening, and to abstain from meat.

At the end of another three days, the patient appeared again and was improving. The treatment was continued for a week, when the salts were discontinued—the rest of the treatment to be as before. By the time three weeks had elapsed, the ulcers were entirely healed, and the size of the limbs was reduced to the natural dimensions. The veins were very much diminished, and the patient could walk without pain or inconvenience—two more weeks completed the cure.

This case is taken from my case book, and dated 1842. I saw the patient three weeks ago, and he has had no return of the disease, but attends to his business as formerly.

